# Swine-Derived Probiotics and Their Metabolites as an Alternative to Veterinary Antibiotics

**DOI:** 10.3390/vetsci12111100

**Published:** 2025-11-18

**Authors:** Mengshi Zhao, Bihong Chen, Song Peng, Guiheng Mei, Meiqin Li, Fengqiang Lin, Tiecheng Sun, Zhaolong Li

**Affiliations:** 1 Institute of Animal Husbandry and Veterinary Medicine, Fujian Academy of Agricultural Sciences, Fuzhou 350013, China; 13375001253@163.com (M.Z.); pengsong@faas.cn (S.P.); 18738371395@163.com (G.M.); linfengqiang@faas.cn (F.L.); 2 Quanzhou Animal Husbandry Station, Quanzhou 362000, China; cbhok2000@126.com; 3 Fujian Provincial Animal Husbandry Station, Fuzhou 350001, China; suntiecheng@126.com

**Keywords:** pig farming, gut microbiota, beneficial microorganism, bioactive compound, feed supplementation

## Abstract

The intensive development of livestock and poultry farming has heavily relied on antibiotics, leading to widespread antimicrobial resistance and posing serious threats to food safety and public health. As the industry transitions towards reduced antibiotic use and sustainable animal production, probiotics and their metabolites have garnered attention as functional alternatives. Probiotics are typically administered in the form of microecological preparations by mixing them into feed or water, offering advantages in cost-effectiveness and ease of use, with demonstrated efficacy in promoting animal health. Swine-derived probiotics, in particular, demonstrate host-specific advantages due to their natural adaptation to the porcine gastrointestinal environment, which improves intestinal colonization, pathogen inhibition, and immune modulation. Their metabolites, including short-chain fatty acids, bacteriocins, and exopolysaccharides, further contribute to these benefits through antimicrobial, anti-inflammatory, and barrier-strengthening effects. Recent studies have demonstrated improvements in average daily gain (18–22%) and feed conversion ratio (12–15%), along with a reduction in diarrhea incidence (up to 40–45%) in weaned piglets supplemented with certain probiotic consortia. It should be noted, however, that part of the supporting evidence is derived from in vitro or non-porcine models, and practical outcomes in swine may vary depending on husbandry conditions, probiotic strain, and husbandry conditions. This review systematically summarizes the isolation and identification of swine-derived probiotics, the active components and functions of their metabolites, and the mechanisms of action and application effects of these metabolites as antibiotic-alternative feed additives. It primarily focuses on innovative research advances in probiotic metabolites for enhancing antibacterial activity and improving pig growth performance. Furthermore, the review discusses the prospects for commercial applications and future research directions, aiming to provide theoretical foundations and technical references for green and healthy farming practices.

## Simple Summary

Prompted by the need to combat antibiotic overuse-induced bacterial resistance in livestock farming—a serious threat to food safety and public health—probiotics and their metabolites have emerged as vital alternatives, proving effective in enhancing animal growth, immunity, and gut microecological balance when administered via feed, water, or fermentation. Swine-derived strains show particular promise due to their host compatibility, pathogen-inhibiting potential, and cost-effectiveness. This review systematically analyzes their isolation, metabolite functions, mechanisms of action, and application efficacy while evaluating innovations and future prospects to advance sustainable, antibiotic-free animal farming.

## 1. Probiotics and Their Beneficial Effects

Antibiotics, as crucial agents in the prevention and treatment of bacterial infections, are wildely utilized in animal husbandry [1]. However, their extensive application has resulted in significant threats, including the dissemination of antibiotic resistance genes, disruption of intestinal microbiota, and environmental pollution [2]. In global swine production, the prolonged and excessive use of antibiotics has facilitated the spread of resistant bacteria and increased drug residues, threatening both product safety and public health [3]. Consequently, many regions are implementing stricter regulations. For instance, the European Union has long enforced restrictions on antibiotic growth promoters, while market pressures in the United States and Brazil are also prompting a shift toward reduced usage. In China, the “Feed Antibiotic Ban” (Announcement No. 194, 2019) officially prohibited the use of antibiotic feed additives as of July 2020, accelerating the need for safe and effective antibiotic alternatives (AAs) [4].

**Figure 1:**
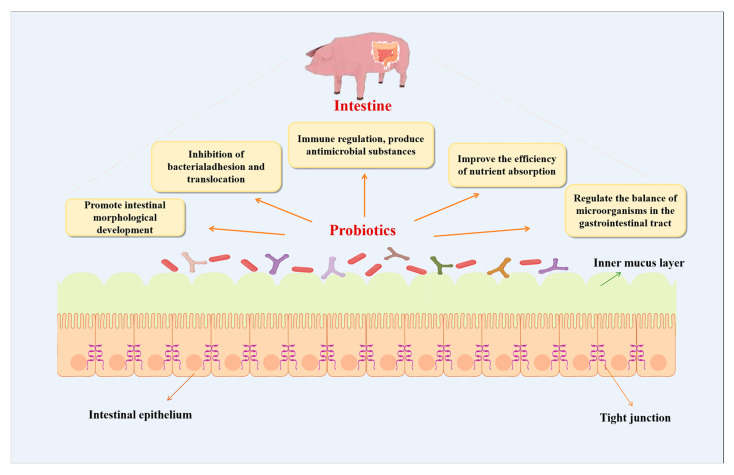
Probiotics and their beneficial effects through multiple mechanisms.

Various AAs candidates, including enzymes, plant extracts, acidifiers, and probiotics, as well as probiotics and their metabolites, have received increasing attention as functional feed additives [5]. Probiotics are live microorganisms conferring health benefits to the host and are commonly applied in animal husbandry as feed additives [6,7]. Major genera include *Bifidobacterium*, *Lactobacillus*, and *Bacillus* (Table 1) [8]. Their beneficial effects are mediated through multiple mechanisms, including nutrient digestion, immune modulation, intestinal development, and microecological maintenance (Figure 1). Under experimental conditions, certain probiotic supplements have been shown to reduce diarrhea incidence by 30–40% and improve the feed conversion ratio by 5–10% in weaned piglets [9,10].

In swine production, swine-derived probiotics, as indigenous commensals, offer distinct advantages over exogenous strains. Beneficial microorganisms isolated from healthy pigs, such as lactic acid bacteria, bacilli, and yeasts, typically exhibit superior gut colonization capacity [25,26]. This enhanced colonization not only competitively excludes opportunistic pathogens but also strengthens the host’s resistance to infections, a mechanism referred to as colonization resistance. For example, certain swine-derived *Lactobacillus* strains have been demonstrated to reduce *E. coli* colonization by 2–3 log units in challenged piglets [27]. Furthermore, swine-derived probiotics produce various bioactive substances through fermentation metabolism, including short-chain fatty acids (SCFAs), antimicrobial peptides, and exopolysaccharides [28]. Owing to their unique value in maintaining intestinal microecological homeostasis, inhibiting pathogen colonization and growth, and enhancing host immune defense functions, swine-derived probiotics and their metabolites have become crucial targets for developing novel AAs strategies [29,30].

Metabolites derived from single strains often exhibit limitations such as a narrow activity spectrum or insufficient potency, and their industrial-scale production is constrained by fermentation process parameters. In recent years, consortia-based synergistic fermentation technology has garnered significant attention due to the diversity of metabolic pathways and the functional synergy and complementarity among strains [31]. This article systematically elucidates the isolation and identification of swine-derived probiotics, the active components and functions of their metabolites, the mechanisms of action, and application efficacy of AAs feed additives based on swine-derived probiotics and their metabolites, and reviews cutting-edge research advances in this field. The goal is to provide a robust theoretical foundation for the development of novel and highly efficient AAs feed additives.

## 2. Isolation, Identification and Selection of Swine-Derived Beneficial Strains

The isolation and identification of swine-derived beneficial strains form the foundation for developing novel probiotic agents and AAs. This approach combines conventional microbiological methods with advanced omics technologies, offering both substantial scientific challenges and application value.

**Figure 2:**
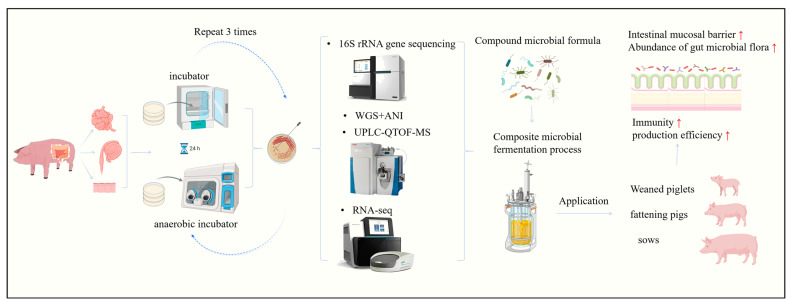
Isolation and Identification of swine-derived Beneficial Strains. Note: The red arrow indicates an increase.

The standard isolation procedure typically begins with the aseptic collection of gastrointestinal contents, fecal samples, or colostrum from healthy pigs (Figure 2). Strains isolated from colostrum (*lactobacilli* and *bifidobacteria*) typically exhibit superior capabilities in gut colonization and immune regulation. In contrast, fecal-derived strains (*butyrate-producing clostridia*) are generally more adept at dietary fiber decomposition, competitive exclusion of pathogens, and tolerance to digestive stresses [32]. Following collection, samples undergo serial dilution in sterile phosphate-buffered saline (PBS) before being evenly spread onto selective solid media. These plates are subsequently incubated in an anaerobic workstation (oxygen concentration < 0.1%) at 37 °C for 48–72 h to accurately simulate the intestinal anaerobic microenvironment. This approach significantly enhances target strain enrichment efficiency, yielding a 3- to 5-fold increase compared to conventional methods [33,34]. Following incubation, individual colonies are selected and purified through three consecutive streak-plating generations on fresh media to ensure genetic homogeneity.

While these classical culture-based techniques remain foundational for probiotic isolation, recent methodological advances have greatly expanded the resolution and scope of strain characterization through multi-omics integration. Current identification technologies demonstrate a trend toward multidimensional integration. The combination of traditional physiological-biochemical characterization with 16S rRNA gene sequencing enables preliminary taxonomic classification at the phylum/genus level [35,36]. However, its resolution is limited for distinguishing closely related species due to the high conservation of the 16S rRNA gene. In contrast, whole-genome sequencing (WGS) coupled with average nucleotide identity analysis achieves precise discrimination of closely related strains exhibiting >99% homology, substantially enhancing identification resolution and efficiency [37]. Nevertheless, the cost and bioinformatic demands of WGS can limit its routine use. For functional characterization, metabolomics techniques such as ultra-performance liquid chromatography coupled with quadrupole time-of-flight mass spectrometry (UPLC-QTOF-MS) facilitate systematic identification of strain-specific metabolites such as short-chain fatty acids and vitamins [38]. Similarly, transcriptomics (RNA-seq) elucidates the molecular regulatory networks that underlie stress responses including acid and bile salt tolerance [39]. These multi-omics technologies provide a critical technological underpinning for screening high-performance probiotic strains. By integrating genomic data with phenotypic metabolomic profiles, researchers can select strains based not only on their genetic potential but also on their documented functional output. This approach enables the identification of candidates with enhanced survival, colonization, and metabolic efficacy in the swine gastrointestinal environment.

Following genomic and functional screening, it is imperative to validate the viability and robustness of candidate strains under simulated practical conditions. This step bridges the gap between genetic potential and practical application.

In vitro viability assessments serve as a primary, cost-effective screening tool. Key tests include evaluating tolerance to simulated gastric acid (pH 2.0–3.0) and intestinal bile salts, with survival quantified by plate counts or flow cytometry; assessing thermal stability at feed pelleting temperatures (60–80 °C); and determining storage stability in various carrier matrices. Data from these studies provide critical go/no-go decisions for advancing candidates to in vivo testing.

In vivo studies are indispensable, as in vitro systems cannot fully replicate the complex gastrointestinal environment. These trials involve administering the probiotic and tracking viable cells through fecal samples to confirm their survival and gut transit. Post-euthanasia analysis of digestive contents and mucosal tissues further assesses the strain’s ability to colonize specific niches (ileum, colon) [40]. Crucially, in vivo viability data must be correlated with health and performance outcomes—including gut morphology, immune markers, and growth performance—to establish a direct link between the presence of the viable probiotic and its functional benefits.

## 3. Metabolites Derived from Beneficial Microorganisms of Swine Origin

### 3.1. Short-Chain Fatty Acids (SCFAs)

**Figure 3:**
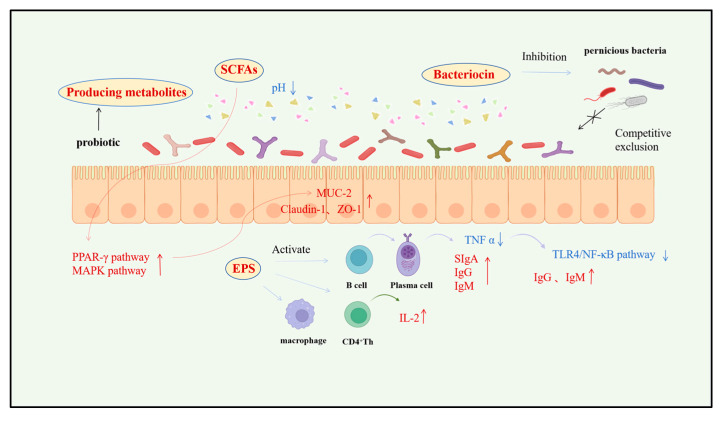
Bioactive Components and Functions of swine-derived Beneficial Strain Metabolites. Note: The red arrow indicates an increase. The blue arrow represents a decrease.

SCFAs, the principal end-products of dietary fiber fermentation by gut microbiota, play a pivotal regulatory role in maintaining intestinal homeostasis and pro-/anti-inflammatory balance in swine [41,42]. Dominant components include acetate, propionate, and butyrate, collectively accounting for approximately 80% of total SCFAs (Table 2). Butyrate plays a particularly significant physiological role as the preferred energy substrate for colonic epithelial cells, being directly absorbed and utilized in mucosal barrier fortification [43,44]. Schilderink [31] demonstrated in cell cultures and rodent models that butyrate significantly upregulates tight junction proteins (Claudin-1 and ZO-1) through histone deacetylase (HDAC) inhibition, thereby enhancing intestinal barrier integrity and reducing pathogen and endotoxin translocation risks (Figure 3).

At the metabolic regulatory level, SCFAs suppress key lipogenesis gene expression via the peroxisome proliferator-activated receptor gamma (PPAR-γ) pathway while synergistically enhancing mineral bioavailability through organic acid chelation, ultimately optimizing growth performance [45,46]. Their immunomodulatory functions include: Promoting regulatory T cell (Treg) differentiation via histone acetylation-mediated epigenetic modification; Polarizing macrophages toward an anti-inflammatory phenotype through G protein-coupled receptor 43 (GPR43) signaling, effectively mitigating stress-induced inflammation in weaned piglets [47]. These mechanisms, while well-documented in murine systems, require further validation in swine.

Furthermore, SCFAs selectively inhibit pathogenic bacteria (*Escherichia coli*) proliferation by reducing intestinal pH, while simultaneously facilitating metabolic cross-feeding with commensals such as *Clostridium butyricum* to restore microbial equilibrium [48,49].

**Table 2:** Mechanisms of action and effects of short-chain fatty acids produced by probiotics.

Compound	Mechanism	Effects
Acetic acid	Acetate serves as an energy source for colonic cells, mucosal epithelial cells, and muscle tissue. It also participates in hepatic metabolism and crosses the blood–brain barrier to regulate appetite through central mechanisms.	The role of short-chain fatty acids in the interplay between gut microbiota and diet in cardio-metabolic health [50]
Propionic acid	Propionate, absorbed in the colon, is primarily used for hepatic gluconeogenesis. It enhances intestinal epithelial activity and suppresses the synthesis of cholesterol and lipids.	Propionate alleviates palmitic acid-induced endoplasmic reticulum stress by enhancing autophagy in calf hepatic cells [51]
Butyric acid	Butyrate acts as the main energy substrate for the colon and cecum. It strengthens the intestinal barrier and exerts anti-inflammatory effects via HDAC inhibition, while also modulating immune cell differentiation.	Short-chain fatty acids and their producing organisms: An overlooked therapy for IBD? [52]
Pentoic acid	Valerate inhibits Th17 cell proliferation and IL-17A production, downregulates key Th17-related genes, and promotes IL-10 expression along with metabolic reprogramming, thereby reducing pathogenicity.	The short-chain fatty acid pentanoate suppresses autoimmunity by modulating the metabolic-epigenetic crosstalk in lymphocytes [53]

### 3.2. Antimicrobial Peptides

Naturally produced antimicrobial compounds derived from swine-derived beneficial strains—such as bacteriocins, organic acids, and defensins—exhibit potent broad-spectrum antimicrobial activity (Table 3) [54,55]. These compounds are proposed to help maintain intestinal microecological homeostasis and defend against pathogen invasion [56,57,58,59]. Among them, bacteriocins, as ribosomally synthesized antimicrobial peptides, demonstrate high target specificity and biosafety [60,61]. Within porcine microbiota, bacteriocins produced by *Lactobacillus* spp. have been most extensively studied. In vitro studies, such as that by Du Kun [62] demonstrated that plantaricin secreted by *Lactobacillus plantarum* disrupts cell membrane integrity and induces transmembrane pore formation, leading to collapse of ion gradients and leakage of macromolecules, thereby effectively inhibiting the proliferation of Gram-negative pathogens such as *Escherichia coli* and *Salmonella* spp. (Figure 3).

Notably, specific bacteriocins can inhibit pathogen biofilm formation by interfering with quorum-sensing systems, potentially blocking their adhesion to intestinal epithelial cells and reducing infection risks [63,64]. As reported by Grande Burgos [65], the cyclic bacteriocin enterocin AS-48 from *Enterococcus* not only effectively inhibits Staphylococcus aureus biofilm development but also suppresses the growth of *Listeria monocytogenes, Salmonella*, and enterotoxigenic *Escherichia coli*.

Compared to conventional antibiotics, bacteriocins may exhibit a lower potential for inducing resistance due to their specific targets. They can be enzymatically degraded by proteases (trypsin) in the gastrointestinal tract without residue accumulation in animal products, establishing them as promising antibiotic alternatives [66,67]. However, their efficacy and stability under commercial swine production conditions require further investigation.

**Table 3:** Common Bioactive Peptides produced by probiotics and their mechanisms of action.

Compound	Mechanism	Effect
Bacteriocins	Proteinaceous toxins that inhibit the growth of closely related bacterial strains and foodborne pathogens by forming pores in the cell membrane, disrupting cell wall synthesis, or inhibiting enzyme activity.	Bacteriocins: Properties and potential use as antimicrobials [68]
Bioactive Peptides	Peptides released during the fermentation of milk, legumes, or other proteins by probiotics. Exhibits antihypertensive (ACE-inhibition), antioxidant, antimicrobial, immunomodulatory, and opioid-like activities.	Bioactive peptides from meat: Current status on production, biological activity, safety, and regulatory framework [69]
Surface Layer Proteins	Crystalline protein structures coating the cell surface. Contribute to adhesion to the intestinal epithelium, competitive exclusion of pathogens, modulation of immune responses, and maintenance of epithelial barrier function.	Lactobacillus surface layer proteins: structure, function and applications [70]

### 3.3. Exopolysaccharides (EPS)

EPS from swine-derived probiotics are proposed to regulate gastrointestinal health and immune function through multiple mechanisms (Figure 3). Their bioactivities encompass antioxidant effects, immunomodulation, intestinal mucosal barrier construction, and microbial homeostasis maintenance(Table 4) [71,72,73]. Recent in vitro studies highlight EPS’s potent in vitro antioxidant activity: it enhances cellular self-defense capacity while effectively scavenging free radicals and mitigating reactive oxygen species (ROS)-induced oxidative damage, thereby preserving cellular structural integrity [74,75].

Regarding immunomodulation, in vitro evidence suggests EPS enhances host–pathogen resistance by activating immune cells including macrophages, B lymphocytes, and T lymphocytes [76,77,78]. This process involves binding to immune cell surface receptors, triggering downstream signaling transduction pathways that promote immune cell activation and functional enhancement, ultimately elevating systemic immune responses [79].

For intestinal mucosal barrier protection, EPS operates through dual mechanisms: interaction with mucins in the intestinal mucus layer to form a physical barrier that inhibits pathogen epithelial adhesion [80], while simultaneously stimulating goblet cells to secrete MUC2 mucin, increasing mucus layer thickness by 2- to 3-fold to block pathogen invasion [81]. It is important to note that “increasing mucus layer thickness by 2- to 3-fold,” are primarily supported by rodent or in vitro studies and require direct validation in swine. In microbial homeostasis regulation, EPS may function as a prebiotic to enhance microbial diversity and promote the proliferation of beneficial bacteria while suppressing the colonization of pathogens such as *Clostridium perfringens*, although data from swine are limited [82,83].

**Table 4:** Common Polysaccharides produced by probiotics and their mechanisms of action.

Compound	Mechanism	Effects
Exopolysaccharides (EPS)	High-molecular-weight sugar polymers secreted into the environment. Form a protective biofilm, aid in adhesion to surfaces, protect the producing bacterium from harsh conditions (acid, bile), and modulate the host immune system towards an anti-inflammatory state.	Exopolysaccharides from probiotic bacteria and their health potential [84]
Lipopolysaccharides (LPS) (from specific probiotic strains)	Component of the outer membrane of Gram-negative bacteria. Probiotic-derived LPS often has a less inflammatory structure (penta-acylated lipid A) that can act as an antagonist to more inflammatory LPS from pathogens, thereby training the immune system and reducing excessive inflammation.	The role of lipopolysaccharide in the development of atopy in humans [85]
Capsular Polysaccharides (CPS)	Tightly bound polysaccharide layer surrounding the cell. Provides physical protection against phagocytosis and antimicrobial peptides, facilitates biofilm formation, and can directly interact with host pattern-recognition receptors to elicit immunomodulatory effects.	The Intestinal Commensal, Bacteroides fragilis, Modulates Host Responses to Viral Infection and Therapy: Lessons for Exploration during Mycobacterium tuberculosis Infection [86]

### 3.4. Vitamins and Coenzymes

Vitamins (including B-complex vitamins and vitamin K) and coenzymes (coenzyme A, NAD^+^) derived from porcine beneficial strain metabolism are thought to enhance host nutritional metabolic efficiency and physiological functions by engaging core metabolic pathways and signaling networks [87,88,89]. Lactobacillus reuteri-synthesized 5-methyltetrahydrofolate (5-MTHF), a highly bioavailable active folate, exhibits 42% higher absorption efficiency mediated by the reduced folate carrier (RFC) compared to synthetic folate, a finding demonstrated in human studies. Its potential to prevent megaloblastic anemia in swine remains to be evaluated [90,91]. Riboflavin (vitamin B_2_), as a precursor of flavin adenine dinucleotide (FAD), increases mitochondrial oxidative phosphorylation efficiency by 18% through activation of the succinate dehydrogenase complex, while simultaneously modulating Th17/Treg cell balance via the aryl hydrocarbon receptor (AHR) signaling axis to exert immunoregulatory functions; however, its efficacy and optimal dosage in swine require further investigation [92]. Menaquinone-7 (MK-7), a vitamin K_2_ component serving as an essential cofactor for γ-glutamyl carboxylase (GGCX), promotes γ-carboxylation of osteocalcin, increasing bone ash content by 8–10% and significantly improving limb-hoof structural integrity in breeding swine [93,94]. Furthermore, the coenzyme nicotinamide adenine dinucleotide (NAD^+^) has been demonstrated in cellular and animal models to enhance intestinal epithelial cell autophagy through SIRT1 pathway activation, thereby mitigating oxidative stress damage; its protective role and mechanism in swine intestinal health warrant further validation [95].

## 4. Mechanisms and Efficacy of Swine-Derived Probiotic Metabolites

Swine-derived probiotic metabolites represent a promising class of natural bioactive compounds that contribute to intestinal health through multidimensional mechanisms including the promotion of morphological development, immune modulation, enhancement of nutrient absorption, metabolic optimization, and maintenance of microbial homeostasis. These integrated functions suggest considerable potential as part of antibiotic-reduction strategies, although their application requires further validation under commercial conditions (Figure 3).

### 4.1. Enhancement of Intestinal Morphological Development and Physical Barrier Function

Probiotic metabolites contribute to the reinforcement of intestinal barrier integrity by promoting the proliferation and differentiation of epithelial cells. Short-chain fatty acids (SCFAs), produced by swine-derived *Lactobacillus*, have been shown to improve jejunal villus development and increase the villus height-to-crypt depth (V/C) ratio, which may enhance nutrient absorption capacity [96,97,98]. For instance, Hao Ding [99] reported increased ileal villus height and V/C ratio in weaned piglets, suggesting a potential expansion of the absorptive surface area.

In terms of mucus barrier enhancement, propionic and butyric acids appear to operate through synergistic mechanisms involving stimulation of goblet cell proliferation and activation of the MAPK pathway, leading to upregulation of MUC2 mucin secretion. Some studies report statistically significant thickening of the mucus layer (*p* < 0.05), which could help inhibit pathogen adhesion [100,101,102]. Additionally, Yue Yingxue [103] observed that butyrate from *Clostridium butyricum* increased occludin expression by 1.8-fold (*p* < 0.01), indicating a strengthening of barrier function.

Furthermore, SCFAs may also modulate intestinal motility via binding to G protein-coupled receptors (GPR41/43), with some swine studies indicating increased peristalsis frequency and reduced chyme transit time, thereby potentially limiting exposure to luminal pathogens [104,105,106,107]. It should be noted, however, that such effects may vary with metabolite concentration and host physiological state.

### 4.2. Immunomodulation and Antimicrobial Substance Production

Swine-derived probiotics and their metabolites exhibit bidirectional immunomodulatory properties. Some studies report elevated serum immunoglobulin (IgM, IgG) and interleukin-2 (IL-2) [108,109], along with increased secretory IgA in intestinal tissues, suggesting enhanced systemic and mucosal immunity [110,111]. For example, Wang [112] observed increased serum immunoglobulins in piglets supplemented with *Clostridium butyricum* and *Enterococcus faecalis*, accompanied by reduced diarrhea incidence. Mizumachi [113] reported that fermented feed from *Lactobacillus plantarum* LQ80 reduced tumor necrosis factor-alpha (TNF-α) levels and increased total serum IgM/IgG concentrations in weaned piglets compared to unfermented diets (Figure 3).

On the other hand, antimicrobial compounds such as bacteriocins EPS may inhibit pathogen adhesion and biofilm formation through membrane perforation (disrupting pathogen membrane integrity to cause ion imbalance and content leakage) and competitive exclusion (occupying intestinal binding sites) [114,115]. Ashlesha Kaushik [116] confirmed that nisin from *Lactococcus lactis* induces membrane perforation in methicillin-resistant Staphylococcus aureus, significantly impairing virulence factor expression. *Bifidobacterial* EPS specifically blocks surface adhesins (fimbriae, flagella) of pathogens, reducing adherence of enteropathogenic *Escherichia coli* to Caco-2 enterocytes by 37–52% [117,118,119]. Beyond organic acids and bacteriocins, hydrogen peroxide produced by lactobacilli and commensal microbiota constitutes a critical antimicrobial mechanism [120,121]. It diminishes pathogen virulence, compromises epithelial invasiveness, or penetrates cells to disrupt transcriptional regulation and signal transduction pathways, ultimately inducing pathogen death [122], though its practical significance in swine gut environments remains investigational.

Furthermore, metabolites from beneficial strains have been shown in some models to alleviate inflammatory signaling, such as the TLR4/NF-kappaB pathway, which is implicated in barrier dysfunction [123]. Yin [124] discovered that metabolites from porcine beneficial strains alleviate dextran sulfate sodium (DSS)-induced colitis and intestinal barrier damage in mice by suppressing TLR4/NF-kappaB inflammatory signaling. Zhang [125] further demonstrated that Lactobacillus plantarum ameliorates *Clostridium* perfringens-induced barrier dysfunction and inflammatory cascades through coordinated regulation of TLR4/NF-kappaB signaling and mitophagy.

### 4.3. Enhanced Nutrient Absorption Efficiency and Metabolic Pathway Optimization

Probiotic metabolites may improve nutrient digestion and absorption efficiency by enhancing digestive enzyme activity, optimizing the intestinal microenvironment, and regulating host metabolic gene expression [126]. Key enzymes such as amylases and proteases produced through their metabolism effectively degrade macromolecular nutrients (starch and proteins) in feed into absorbable micromolecules (glucose and amino acids), potentially increasing the digestibility of crude protein and other nutrients in nursery pigs [127]. Zhou Yuanli [128] demonstrated that feeding *Lactobacillus plantarum*-fermented feed significantly enhanced protease, lipase, and α/β-amylase activities (*p* < 0.05) in intestinal contents of nursery pigs, thereby improving nutrient absorption efficiency and increasing beneficial bacterial abundance (Figure 3).

SCFAs also contribute to metabolic regulation: acetate and propionate participate in lipogenesis and gluconeogenesis, while butyrate serves as an energy source for colonocytes and may activate PPAR-γ signaling, potentially improving metabolic efficiency [129,130]. Tang [131] observed improvements in backfat thickness and lean meat percentage in finishing pigs supplemented with *Clostridium butyricum*.

Furthermore, B vitamins synthesized by probiotics synergize with host metabolic enzymes: vitamin B_12_ mediates methylmalonyl-CoA mutase activity to enhance carbohydrate and lipid metabolism, resulting in a 40% increase (*p* < 0.01) in serum vitamin B_12_ levels in piglets and significant improvements in growth performance indicators such as average daily gain [132].

### 4.4. Modulation of Gastrointestinal Microbial Balance and Intestinal Health Maintenance

Swine-derived beneficial strains and their metabolites establish a stable microecological equilibrium system by targeted regulation of gut microbial community structure and function, suppressing pathogen proliferation while promoting colonization of beneficial bacteria [133,134]. This mechanism constitutes the core foundation for antibiotic-alternative additives to achieve “bacteriotherapy” functionality [135,136], primarily manifested through: SCFAs produced by beneficial bacterial metabolism significantly lowering intestinal pH to create an acidic environment inhibitory to pathogen growth. Such selective pressure directly suppresses replication of pathogenic bacteria including *Escherichia coli* and *Salmonella* spp., while simultaneously stimulating proliferation of beneficial bacteria such as *Bifidobacterium* and *Lactobacillus*, thereby optimizing microbial community structure [137,138].

Bai Peidian [139] demonstrated that feeding compound probiotic preparations significantly increased the relative abundance of Faecalibacterium in piglet cecum (+28.6%, *p* < 0.05) compared to controls, whereas abundances of *Campylobacter* and *Lachnospira* decreased by 19.3% and 14.7%, respectively. This microbial restructuring positively correlated with intestinal health status. Cui Wenzhi [140] observed that dietary supplementation with swine-derived probiotics significantly reduced diarrhea incidence by 36% (*p* < 0.01) in weaned piglets. Further investigations revealed that probiotic microorganisms enhance gut microbiota α-diversity (Shannon index increase: 1.8–2.3), elevate the relative abundance of Firmicutes (+15.4%), and suppress overproliferation of Bacteroidetes and Proteobacteria (decreases of 12.1% and 8.9%, respectively), collectively optimizing microbial architecture and maintaining microecological homeostasis (Figure 3). While these changes suggest improved ecological balance, it is important to note that microbial responses are context-dependent and may vary with diet, environment, and host genotype.

## 5. Application of Swine Beneficial Strains and Their Metabolites as Antibiotic Alternatives

In the field of antibiotic-alternative feed additives, swine-derived *Lactobacillus* demonstrates promising mechanisms, though single-strain probiotics are often limited by challenges in stability and storage viability. Consequently, composite probiotic preparations that leverage synergistic functional advantages have emerged as a more effective strategy [141,142]. Studies indicate that mixed fermentation using multiple swine-derived *Lactobacillus strains* can produce enhanced antimicrobial effects, achieving significantly greater inhibition against pathogens such as *Escherichia coli* and *Salmonella* spp. compared to fermentates from single strains, with some studies reporting increases in inhibition zone diameter of 35–48% (*p* < 0.01) under specific experimental conditions [143,144] (Figure 3). It should be noted that such pronounced effects may vary with experimental design.

From a veterinary standpoint, the strategic application of probiotics is most effective when aligned with the specific physiological challenges and health management goals of each stage of the swine life cycle. Probiotics have shown substantial benefits in both sow and piglet production [145]. Supplementation during gestation and lactation has been found to improve sow body condition, increase milk quality and yield, and enhance immunoglobulin content in colostrum, thereby promoting piglet growth and survival rates [146]. Composite fermented feeds may further enhance nutritional value through microbial metabolism, overcoming species barriers while simultaneously boosting antibacterial activity and growth performance [147].

The weaning period represents a high-stress transition associated with intestinal dysbiosis and increased susceptibility to diarrhea. Probiotic intervention at this stage plays a critical role in alleviating post-weaning stress, supporting the development of intestinal barrier function, and reducing the incidence of digestive disorders. Studies report improvements in the feed conversion ratio (FCR) by 12–15% and average daily gain (ADG) by 18–22% in weaned piglets receiving composites [148,149]. Furthermore, studies have shown that trials with specific composite probiotics have reported a 40–45% reduction in diarrhea incidence and significant improvements in growth metrics, likely mediated through enhanced intestinal development and antioxidant capacity [150].

During the growing and finishing phases, the primary goals shift toward optimizing growth performance and feed conversion. The use of composite probiotics and fermented feeds has been shown to enhance nutrient availability and improve growth metrics [151]. Li Genghui [152] confirmed that fermented feed increased finishing pig average daily gain by 43.5% (*p* < 0.01) and reduced feed: gain ratio by 28.8% compared to conventional feed.

Beyond swine-derived probiotics bacteria, other antibiotic alternatives like yeast-based supplements operate through distinct yet complementary mechanisms. Whereas swine-derived probiotics often directly modulate the gut microbiota and strengthen barrier function, yeast-based alternatives may exert benefits by altering the gut environment. For instance, Cao Di [153] reported that yeast-based antibiotic alternatives in late-gestation and lactating sow diets significantly increased total volatile fatty acids (VFA) in ileal chyme (+32.7%, *p* < 0.05), with butyrate proportion rising by 18.3 percentage points—thereby helping inhibit pathogenic microbial proliferation.

Despite these promising results, the practical application of probiotic additives faces several important challenges beyond technical efficacy. Economic feasibility must be considered, as production costs of composite probiotics—including fermentation, stabilization, and delivery technologies—may be higher than those of conventional additives, though potentially offset by improved performance and reduced veterinary costs. Regulatory aspects also play a critical role, as approval processes for probiotic feed additives vary across regions and may require substantial evidence of safety and efficacy. Additionally, consumer acceptance represents another key factor, with growing preference for natural and antibiotic-free animal products creating market opportunities for probiotic-based solutions [154].

Collectively, composite swine derived from porcine sources show substantial potential as antibiotic alternatives. They may enhance swine growth performance, reproductive efficiency, and health status by improving feed nutritional quality, precisely modulating gut microecology, and strengthening immune function (Table 5). However, their practical application faces several challenges, including production cost, standardization of probiotic consortia, stability during feed processing and storage, and inconsistent responses across different production systems. Future efforts should focus on large-scale validation and addressing these variabilities to fully realize their potential in supporting sustainable livestock production.

## 6. Conclusions

Current findings underscore that composite probiotic additives, by capitalizing on synergistic interactions among microbial strains, offer superior efficacy compared to single-strain formulations, marking a clear direction for future development in this field. Evidence indicates that incorporating swine-derived fermented feed into piglet diets contributes to notable health and productivity improvements, including reduced diarrhea incidence, enhanced growth performance and feed efficiency, as well as elevated serum immunoglobulin concentrations. However, several technical challenges remain, particularly in maintaining strain viability, standardizing fermentation protocols, and establishing consistent quality control systems for such fermented products.

Future research should efforts should focus on: (1) Elucidating the molecular mechanisms underlying bacterial metabolite interactions with host physiology; (2) Optimizing high-density fermentation parameters to enhance yield and functionality; (3) Developing scientific quality evaluation frameworks for standardized product assessment; and (4) integrating probiotics with precision nutrition approaches and other antibiotic alternatives, such as phytogenics and organic acids, for synergistic effects. Furthermore, long-term field studies are essential to comprehensively validate efficacy, safety, and economic benefits in diverse production systems. By addressing these challenges and research priorities, swine-derived probiotics and fermented feeds can advance toward sustainable, large-scale implementation in swine production, contributing to reduced antibiotic dependence and improved animal health management.

## Figures and Tables

**Table 1:** Bacteria that have demonstrated probiotic effects in the veterinary field.

	Genus	Species	Effect on Animal Health
Bacteria	*Lactobacillus*	*L. acidophilus*	A core probiotic species primarily residing in the small intestine; supports digestive health [11].
		*L. casei*	Widely used in fermented foods; supports gut microbiota balance [12].
		*L. paracasei*	Commonly researched for immune support [13].
		*L. rhamnosus*	One of the most clinically documented strains; effective against antibiotic-associated diarrhea [14].
		*L. plantarum*	Highly adaptable; often studied for managing IBS symptoms [15].
	*Bifidobacterium*	*B. animalis* subsp. *lactis*	Extremely common in dairy products; supports immune and digestive health [16].
		*B. longum* subsp. *longum*	A dominant inhabitant of the adult gut; researched for IBS and anti-inflammatory effects [17].
		*B. breve*	Prevalent in infants; studied for the prevention of necrotizing enterocolitis (NEC) [18].
	*Bacillus*	*B. coagulans*	Spore-forming bacterium; highly stable and resistant to heat and acid [19].
		*B. subtilis*	Spore-forming; used for its enzymatic activity and support for protein digestion and gut health [20].
		*B. clausii*	Spore-forming; widely used, especially in Europe, for preventing antibiotic-associated diarrhea [21].
	*Enterococcus*	*E. faecium*	Used for management of diarrhea. Note: Some strains are vancomycin-resistant (VRE), so careful selection is critical [22].
fungus	*Saccharomyces*	*S. boulardii*	The most well-known probiotic yeast; effective against antibiotics [23].
	*Kluyveromyces*	*K. marxianus*	Used in dairy fermentations; also researched for its potential probiotic properties in gut health [24].

**Table 5:** A comparison of the therapeutic effects of probiotics and antibiotics.

Disease/Disorder	Target Population/Species	Intervention (Dosage/Duration)	Key Efficacy Outcomes & Mechanisms
Necrotic Enteritis	Livestock: Broiler chickens (Poultry).	Antibiotic: Ionophores (Salinomycin) or therapeutic antibiotics (Bacitracin). Probiotic: *Bacillus subtilis* or *Clostridium butyricum* spores. ~1–5 × 10^5^ CFU/g of feed. Duration: Throughout the grow-out period.	Antibiotic: Effective for prevention and treatment but faces resistance and withdrawal issues. Probiotic: Reduces mortality and intestinal lesions caused by *Clostridium perfringens*. Mechanism: Competitive exclusion, production of antimicrobial compounds (bacteriocins), stimulation of immune responses [137].
Acute Infectious Diarrhea	Humans: Infants & Children. Livestock: Neonatal piglets.	Antibiotic: Only for bacterial causes (e.g., *E. coli* scours). Probiotic: *L. rhamnosus* GG or *Bacillus clausii*. ~1–10 Billion CFU/day for 5–7 days.	Antibiotic: Targets specific pathogens but may disrupt microbiota. Probiotic: Shortens duration of diarrhea. Mechanism: Inhibition of pathogen adhesion, secretion of antibacterial substances, support of mucosal immunity [59].
Post-Weaning Diarrhea	Livestock: Weaned piglets.	Antibiotic: ZnO (pharmacological dose) is widely used, but being phased out in the EU. Therapeutic antibiotics (Colistin). Probiotic: *Enterococcus faecium* or *Bacillus licheniformis*. ~1–5 × 10^9^ CFU/kg feed. Duration: 2–4 weeks post-weaning.	Antibiotic: Effective but contributes to antimicrobial resistance (AMR). Probiotic: Improves growth performance and reduces diarrhea incidence. Mechanism: Stabilizes gut microbiota during stress, enhances digestive enzyme activity, improves gut barrier integrity [101].
Helicobacter pylori Infection	Humans: Infected adults.	Antibiotic: Standard triple/quadruple therapy (PPI + Amoxicillin + Clarithromycin). Probiotic: As adjunct: Specific strains of *Lactobacillus* and *Bifidobacterium*. ~10–20 Billion CFU/day.	Antibiotic: Eradicates pathogen but has high side effect rate. Probiotic: Increases eradication rates and reduces antibiotic side effects (especially diarrhea). Mechanism: May inhibit Hp adhesion, modulate local immune response [138].
Mastitis	Livestock: Dairy cows.	Antibiotic: Intramammary infusions (Cephapirin) during the dry period or for treatment. Probiotic: Intramammary infusions of *lactic acid bacteria* (e.g., *Lactococcus lactis*) or oral administration.	Antibiotic: Standard treatment but leads to milk discard periods. Probiotic: Promising for prevention and treatment, reducing somatic cell count (SCC). Mechanism: Competitive exclusion of pathogens like *S. aureus*, modulation of the local immune response in the mammary gland [137].
General Growth Promotion/Health Maintenance	Livestock: Poultry and Swine.	Antibiotic: Growth-Promoting Antibiotics (AGPs)—now banned/restricted in many regions. Probiotic: Various (*Bacillus*, *Lactobacillus*, *Enterococcus strains*). ~0.5–2 × 10^9^ CFU/kg feed. Duration: Continuous.	Antibiotic: Historically improved growth rate and feed efficiency. Probiotic: Modestly improves Average Daily Gain (ADG) and Feed Conversion Ratio (FCR). Mechanism: Competitive exclusion of pathogens, improved nutrient digestibility, enhanced intestinal health and morphology [126].

## Data Availability

No new data were created or analyzed in this study. Data sharing is not applicable to this article.
